# Perceived Severity of Stressors in the Intensive Care Unit: A Systematic Review and Semi-Quantitative Analysis of the Literature on the Perspectives of Patients, Health Care Providers and Relatives

**DOI:** 10.3390/jcm10173928

**Published:** 2021-08-31

**Authors:** Henning Krampe, Claudia Denke, Jakob Gülden, Vivian-Marie Mauersberger, Lukas Ehlen, Elena Schönthaler, Maximilian Markus Wunderlich, Alawi Lütz, Felix Balzer, Björn Weiss, Claudia D. Spies

**Affiliations:** 1 Department of Anesthesiology and Operative Intensive Care Medicine (CCM, CVK), Charité - Universitätsmedizin Berlin, Corporate Member of Freie Universität Berlin, Humboldt-Universität zu Berlin, and Berlin Institute of Health, 13353 Berlin, Germany; henning.krampe@charite.de (H.K.); claudia.denke@charite.de (C.D.); jakob.guelden@charite.de (J.G.); vivian-marie.mauersberger@charite.de (V.-M.M.); lukas.ehlen@charite.de (L.E.); alawi.luetz@charite.de (A.L.); bjoern.weiss@charite.de (B.W.); 2 Institute of Psychology, University of Graz, 8010 Graz, Austria; elena.schoenthaler@medunigraz.at; 3 Institute of Medical Informatics, Charité - Universitätsmedizin Berlin, Corporate Member of Freie Universität Berlin, Humboldt-Universität zu Berlin, and Berlin Institute of Health, 10117 Berlin, Germany; maximilian-markus.wunderlich@charite.de (M.M.W.); felix.balzer@charite.de (F.B.); 4 Department of Healthcare Management, Technische Universität Berlin, 10623 Berlin, Germany

**Keywords:** ICU experience, intensive care, perceived stress, stressor, stressful experience

## Abstract

The aim of this study was to synthesize quantitative research that identified ranking lists of the most severe stressors of patients in the intensive care unit, as perceived by patients, relatives, and health care professionals (HCP). We conducted a systematic literature search in PubMed, MEDLINE, EMBASE, PsycInfo, CINAHL, and Cochrane Library from 1989 to 15 May 2020. Data were analyzed with descriptive and semi-quantitative methods to yield summarizing ranking lists of the most severe stressors. We synthesized the results of 42 prospective cross-sectional observational studies from different international regions. All investigations had assessed patient ratings. Thirteen studies also measured HCP ratings, and four studies included ratings of relatives. Data indicated that patients rate the severity of stressors lower than HCPs and relatives do. Out of all ranking lists, we extracted 137 stressor items that were most frequently ranked among the most severe stressors. After allocation to four domains, a group of clinical ICU experts sorted these stressors with good to excellent agreement according to their stress levels. Our results may contribute to improve HCPs’ and relatives’ understanding of patients’ perceptions of stressors in the ICU. The synthesized stressor rankings can be used for the development of new assessment instruments of stressors.

## 1. Introduction

Critical illness is known to be highly stressful, and for many patients, treatment in an intensive care unit (ICU) constitutes a traumatic experience [1]. There is extensive research available on stressors and stressful experiences of patients in the ICU. Major findings have shown that the stressful experiences of ICU patients are multifactorial. Among the main causes are treatment procedures, physical stressors, illness-related impairments, environmental factors, as well as associated mental distress [1,2,3,4,5,6,7]. Perceived stress can by itself be regarded as a negative patient reported outcome [1,8,9,10]. Moreover, it is associated with worse mental health and cognitive outcomes during and after intensive care, including the respective domains of the post-intensive care syndrome (PICS) [5,10,11,12,13,14,15,16,17]. Thus, research into the perceived severity of stressors is a prerequisite for the development of interventions to reduce stressful experiences of patients, with the long-term objective of enhancing recovery after intensive care. The measurement of ICU-related stressors is diverse. While several researchers developed study-specific measures (e.g., [5,18,19]), many investigations used the *Intensive Care Unit Environmental Stressor Scale* (ICUESS) [3] and its extended adaptation, the *Environmental Stressor Questionnaire* (ESQ) [4]. These standardized questionnaires assess the perceived stress levels of specific stressors with 42 and 50 stressor items, respectively. There are older literature reviews on perceived stressors in the ICU [20,21,22] and brief literature overviews as part of clinical studies (e.g., [8,23,24]). A recent systematic review focused on a rather selected sample of original articles on patient experiences in the ICU [25], and a master thesis conducted a meta-analysis of a restricted sample of nine original studies applying the ICUESS or the ESQ [26]. Taken together, these reviews found that the identification of the most severe perceived stressors has been an important objective of ICU stress research over the last four decades. The studies’ samples, settings, measurements, and results varied, and no homogenous patterns of either stressor experience and/or stress level perception have been identified.

Some studies compared patients’ perceptions of stressors with the ratings of stressors by health care providers (HCP) working in ICUs and/or relatives (e.g., [3,4,8,23]). Their results suggest that both HCPs and relatives rate the severity of patients’ stressors higher than patients do. The majority of studies follows a quantitative approach, but also qualitative and mixed methods have been applied (e.g., [9,27,28,29]).

To our knowledge, recent systematic reviews of quantitative studies on perceived severity of stressors in ICUs are lacking. Quantitative research has used many different outcome instruments to measure subjective stressful experiences of ICU treatment. In order to encompass the diversity of stressors that have been assessed by various instruments, the systematic review at hand is not restricted to the most established questionnaires such as the ICUESS and ESQ. The inclusion of several stressor measures increases the diversity of potentially relevant stressors, but it also contributes to higher data heterogeneity. In this case, classical meta-analytic methods seem less promising because data homogeneity is among the most important methodological prerequisites of sound meta-analytically synthesized results [30]. Thus, a descriptive and semi-quantitative analysis is a more adequate methodological approach.

The main objective of this systematic literature review was to synthesize quantitative research that reports results on ranking lists of the most severe perceived stressors of patients treated in ICUs. Specific objectives were: (1) to descriptively extract data of studies on both specific patient-rated stressor ranking lists and on comparisons of stressor rankings made by patients, HCPs, and relatives; (2) to analyze the extracted data with semi-quantitative methods including a modified Q sort approach to yield summarizing ranking lists of the most severe specific stressors; and (3) to summarize study results on the comparison of general stressor severity ratings of patients, HCPs, and relatives.

## 2. Materials and Methods

### 2.1. Criteria for Considering Studies of This Review

This systematic review focused on quantitative investigations of perceived severity of specific stressors in the ICU by compiling ranking lists of the most severe stressors.

*Types of studies:* We included quantitative original studies that were written in English and were published as articles in scientific peer reviewed journals. We excluded studies with sample sizes below 15 (to avoid biased quantitative results), studies using exclusively qualitative analysis methods, as well as literature reviews, meta-analyses, editorials, commentaries, primary analyses of clinical trials comparing interventions, abstracts of conference papers, and doctoral/master theses.

*Types of participants:* Inclusion criteria for participants comprised patients who were treated in an ICU for more than 24 h and who had a minimum age of 14 years. Studies with samples of relatives of ICU patients, as defined by the specific study design, and studies with samples of HCPs working in ICUs were only considered when an additional patient sample was investigated. We excluded studies from pediatric intensive care.

*Types of outcomes:* We included outcomes regarding the perceived severity of specific stressors or stressful experiences of ICU patients, as rated by patients, their relatives, and ICU HCPs. Only studies that identified at least one ranking list of stress levels of a minimum of five stressors were accepted. As outcome measurement instruments, we allowed standardized questionnaires, single item questions, and structured and semi-structured interviews. As methods of severity rankings, we accepted ranking according to mean item scores, to percent of agreement responses to specific items, as well as rankings made with the Q sort technique, i.e., direct sorting of items regarding a specific criterion, here sorting of stressors regarding their severity. We excluded outcomes that were generated by qualitative analyses of answers to open-ended questions, or outcomes that were restricted to measurements of perceived general stressor severity, such as general stress sum scale scores and/or subscale scores, without identifying rankings of at least five perceived specific stressors. We also excluded studies that exclusively investigated objective stressors in the ICU, as well as studies on mental distress, psychiatric symptoms, and health-related quality of life that did not additionally assess severity of at least five specific perceived stressors.

### 2.2. Search Methods for Identification of Studies

*Electronic searches:* We conducted systematic literature searches in the databases PubMed, MEDLINE, EMBASE, PsycInfo, CINAHL, and Cochrane Library from 1981 to 15 May 2020. On the 15th of May 2020, we ran the final search. *Searching other resources:* Additional searches were carried out in the references of identified papers, as well as in Google citations of papers applying the well-established ICU stressor measures ICUESS [3], ESQ [4], and Recollection Questionnaire [5]. *Search terms and selection of studies:* For searches in titles and abstracts, we used the following search terms: (1) ‘stressors’, AND ‘intensive care’, AND ‘patients’. (2) ‘stress’, AND ‘perception’, AND ‘intensive care’, AND ‘patients’. For searches restricted to titles, we used the following terms: (1) ‘experiences’ OR ‘experience’, AND ‘intensive care’ AND ‘patients’. (2) ‘perceptions’ OR ‘perception’, AND ‘intensive care’ AND ‘patients’.

**Figure 1:**
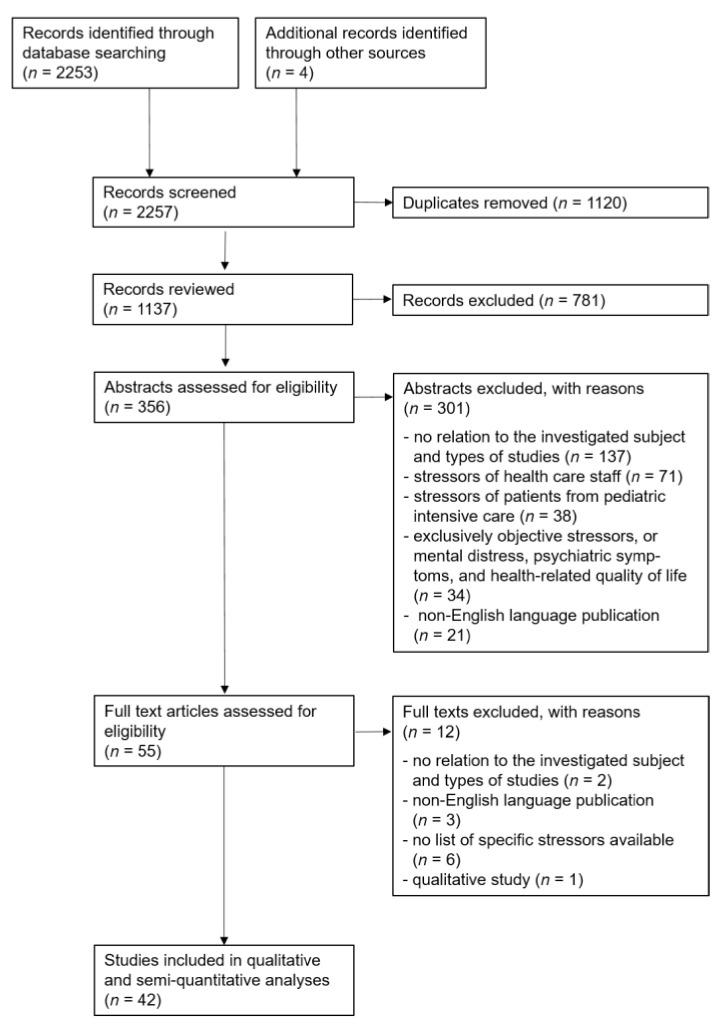
PRISMA flowchart.

Combining hits of all search queries yielded a total of 2257 results. Figure 1 shows the flowchart of the Preferred Reporting Items for Systematic Reviews and Meta-analyses (PRISMA). Three researchers (LE, JG, HK) independently screened the titles and abstracts of these results and selected studies applying the above-mentioned inclusion and exclusion criteria. In case of inconsistent selection results, all three researchers read the abstracts and full texts of the respective studies and discussed their perspectives to find consensus agreement. Out of the 2257 articles that were found in the database searches, we identified 42 quantitative studies that investigated perceived severity of specific stressors or stressful experiences of ICU patients by identifying at least 1 ranking list of stress levels of a minimum of 5 stressors. The major exclusion criteria that we applied comprised studies with no relation to the investigated subject and types of studies (38.5%), stressors of health care staff (19.9%), stressors of patients from pediatric intensive care (10.7%), exclusively objective stressors, or mental distress, psychiatric symptoms, and health-related quality of life (9.5%), as well as non-English language publications (5.9%).

### 2.3. Extraction, Management, and Analyses of Data

*Extraction and management of data:* One author (HK) read the full texts of the 42 identified studies. He extracted study characteristics including data on authors, year of publication, country, study design, number of study centers, sample sizes, types of ICU’s, types of participants (patients, relatives, HCPs), age and gender, clinical characteristics of patients, professional categories of HCPs, measurement instruments of stressors, setting and time of data collection, as well as indicators of psychometric quality of stressor measures. Another author (ES) also read the 42 studies and checked all extracted data for potential errors. In case of disagreement, the two authors conducted consensus discussions to find a common agreement.

Two major outcomes were extracted: (1) severity ranking lists of specific stressors, and (2) comparisons of general severity of stressors, as perceived by patients, relatives, and HCPs (Supplementary Table S1). During a first inspection of the study results, we found that several studies only reported ranking lists of 4 to 10 items, whereas others reported up to 50 items. Thus, we chose to perform a balanced extraction procedure, with at least one ranking list of a minimum of 5 stressors, and a limitation of extracted stressors to a maximum of 10 ranks per ranking list. In case of unique ranks, the criterion number of 10 ranks covers exactly 10 items, which is 1/4 of the ICUESS items and 1/5 of the ESQ items. In case of shared ranks, more than 10 stressors were included to reach the maximum number of 10 ranks. With this procedure, we balanced the two tendencies to, on the one hand, include too much irrelevant items and, on the other hand, to prematurely exclude possibly important items. When studies provided two or more lists on different stressors, all lists were included. In cases of non-English-speaking assessments that had used the ICUESS or ESQ, we replaced incorrectly re-translated item formulations by the original English formulations. In cases of culturally adapted items, we kept the wordings of the respective articles.

*Data analyses:* Data analyses were conducted in a semi-quantitative way. Study characteristics were initially reported descriptively per study, and then summarized with descriptive statistics (number [percent], median [minimum, maximum, interquartile range]. The analyses of comparisons between general stressor ratings of patients, HCPs, and relatives were also carried out descriptively, by way of a simple box-count method. The ana-lyses of stressor rankings were performed in several steps: After the extraction of the stressor ranking lists of all 42 included studies, we pooled the stressors of all studies in three groups: (1) patient ratings, (2) HCP ratings, and (3) ratings of relatives. In a next step, we checked whether there were stressors in the stressor lists of HCPs and relatives that were not yet included in the stressor lists of the patients. Both HCPs and relatives reported only stressors that were also reported by patients. As a consequence, we decided to perform the further semi-quantitative analyses of ranking lists only for the patients’ ratings. Four researchers (CD, LE, JG, HK) removed duplicates and items that were semantically excessively redundant. Next, two researchers (CD, HK) grouped the items according to four domains: (1) Physical, treatment, and disease-related stressors; (2) Mental health; (3) Communication; (4) Environment (Supplemental Table S2). In order to keep domain-specific items in the analyses, the following expert ratings of stressor severity were conducted separately for the four domains. Four experts with ample clinical experience of ICU treatment (one nurse, one psychologist, and two physicians; CD, VM, JG, LE, so two women and two men) sorted the stressors per domain with an adapted Q sort approach [2,31,32]. The sorting criterion was: *‘Rate a given domain-specific stressor in relation to the other domain specific stressors with regard to the severity of stress that it causes for patients who are acutely treated in an ICU’.* The raters each received four envelopes containing cards with the printed names of the domain-specific extracted stressors. Each envelope referred to one of the four stressor domains. The raters were instructed to sort the items per domain. Per instruction, they were asked to allocate one item to one rank, but, when necessary, they were also allowed to build shared ranks with no limits of items per rank. We determined agreement among raters with the intraclass correlation coefficient (ICC), a measure of inter-rater reliability. We calculated ICCs (two-way mixed effects, consistency, multiple raters) between the raters for all four stressor domains. After analyses of inter-rater reliability, we summarized the Q sort data by calculating the mean ranks per item of the four ICU expert raters.

## 3. Results

### 3.1. Study Characteristics

All of the included 42 investigations were prospective cross-sectional quantitative observational studies. Table 1 shows the major study characteristics.

*Study origin and sample sizes:* The included studies were performed in different international regions. Eleven (26.2%) studies were conducted in North America, nine (21.4%) in Europe, eight (19.0%) in Asia—Far East, seven (16.7%) in Asia—Middle East, six (14.3%) in South America, and one (2.4%) in Africa. Since two studies had included two patient samples [33,34], sample size data were available from 44 samples. The median patient sample size was n = 76.5 (min: 16, max: 868, IQR 40.8–113.5). Moreover, 13 studies had also included HCPs [3,4,8,18,23,33,35,36,37,38,39,40,41], with a median sample size of n = 55 in 14 HCPs samples (min: 21, max: 152, IQR 33.8–72.0). There were only four investigations that had included relatives. Mistraletti et al. (2018) recruited two samples of relatives [36], resulting in the availability of sample size data from five samples of relatives: n = 29 and n = 33 [36], n = 30 [35], n = 50 [8], n = 60 [42].

*Age and gender of patients:* A total of 35 studies reported data on patient samples with an average age of 37. The median of the 37 indicators for average age was 55.7 years (min: 38.1, max: 69.4, IQR: 53.0–59.6). A total of 40 studies provided data on gender from 42 patient samples. The median percentages of men and women were 64% (min: 28.3, max: 81.3, IQR: 53.8–70.2) and 36% (min: 18.8, max: 71.7, IQR: 29.8–46.2), respectively. Table 1 shows that data on medical characteristics of patient samples were rather sparse and heterogeneous. Thus, we did not calculate summarizing statistics.

*Age and gender of relatives:* Only two articles reported the average ages of three samples of relatives: 47.0 years and 51.0 years [36], as well as 41.9 years [42]. Four articles provided data on the gender distribution of five samples of relatives. The ratios of men to women were 37.0 %/63.0 % [35], 58.6 %/41.4 % and 48.5 %/51.5 % [36], 22.0 %/78.0 % [8], as well as 37.0 %/63.0 % [42].

*Age, gender, and professions of HCPs:* Six articles provided data on the average age of seven HCPs samples: 30.0 [4], 30.32 [40], 30.6 [41], 33.0 [36], 33.5 and 39.7 [33], as well as 40.1 years [23]. Eleven articles reported gender distribution data of twelve HCPs samples [3,8,18,23,33,35,36,37,38,39,41]. The median percentages of men and women were of 24.3 % (min: 0.20, max: 57.1, IQR: 12.3–46.5) and 68.4 % (min: 0.8, max: 92.5, IQR: 46.3–85.3), respectively. All of the 13 articles that had included HCPs provided data on professions of HCPs [3,4,8,18,23,33,35,36,37,38,39,40,41]. In 10 out of 14 samples, the percentage of nurses (including nurse assistants) was 100% [3,4,18,33,35,37,38,39,40,41]. The rates of nurses were 0% [33], 28.6% [33], 80.0% [8], and 85.0% [23] in the remaining four studies, resulting in a median percentage of nurses of 100% (min: 0, max: 100, IQR: 83.8–100). Only three investigations included physicians, with 100% [33], 28.6% [36], and 8.0% [8], as well as other health care professions, with 12.0% [8], 13.0% [23], and 42.8% [36].

**Table 1:** Characteristics of 42 studies on rankings of perceived intensity of stressors in the ICU (abbreviations see table footer).

Author, Year, Country	Number of SCs; P, HCP, R; Sample Size; Age (Years), m/f; Clinical Characteristics	Instrument, Setting, and Time of Data Collection; Availability of Data on PQ
Abuatiq 2015, USA [23]	**SC**: 1; **P:** n = 70, 58.9 y, 38/32; various diagnoses; medical and surgical ICUs; **HCP:** n = 70, 40.1 y, 14/56; 74% nurses, 11% nurse assistants, 11% respiratory therapists, 3% others.	**P:** 50-item ESQ, during ICU stay; **HCP**: 50-item ESQ, ICU shift; **PQ~**.
Alasad et al., 2015, Jordan [43]	**SC:** 3; **P:** n = 98, 45.7 y, 60/38; no data on diagnoses; medical and surgical ICUs from 3 hospitals.	**P:** 31-item ICEQ, structured interviews, on ward, within 72 h after transfer from ICU; **PQ~**.
Azizi-Fini et al., 2017, Iran [41]	**SC:** 1; **P:** n = 120, 45.6 y, 34/86; no data on diagnoses; no data on types of ICU and mode of admission; **HCP:** n = 60 nurses, 30.6 y, 33/27.	**P, HCP:** Iranian adaption of 50-item ESQ; ^(a)^ **P:** at ICU, at least 48 h after admission, self-report and semi-structured interview; **HCP**: at ICU, self-report; **PQ−**.
Ballard 1981, USA [2]	**SC:** 1; **P:** n = 22, 55.3 y, 15/7; no data on diagnoses; surgical ICU, various surgical procedures, general and urology surgery.	**P:** 40-item Q-sort cards of ICUESS items, 2–3 days after ICU stay; **PQ~**.
Bergbom-Engbert & Haljamae 1989, Sweden [44]	**SC:** 1; **P:** n = 158, 53.3 y, 101/57; various unspecified diagnoses; 2 ICUs (surgical, trauma) from a general hospital; all patients had major surgeries and were mechanically ventilated.	**P:** Structured telephone interview, no name, at median 1.7 years after ICU stay (2 months to 4 years); **PQ~**.
Bernal Ruiz et al., 2018, 2016, Columbia [45,46]	**SC:** 1; **P:** n = 353, 61.8 y, 240/113; 52.9% coronary, 30.3% surgery, and 2.5% medical ICUs of a 4th level health institution; admissions: 81.3% urgent, 18.1% planned, 0.6% other; no data on diagnoses.	**P:** 45-item *‘Escala de Factores Estresantes Experimen-tados por los Pacientes en UCI*’; structured interview, 1–3 days after discharge from ICU, at ward; **PQ+**.
Biancofiore et al., 2005, Italy [33]	**SC:** 1; **P_1_:** n = 104, 50.9 y, 69/35; liver transplant recipients; various diagnoses; **P_2_**: n = 103, 58.0 y, 82/21; major abdominal surgery patients, various diagnoses; P_1_ and P_2_ at the same postsurgical ICU; **HCP_1_:** n = 35 nurses, 33.5 y, 10/25. **HCP_2_**: n = 21 physicians, 39.7 y, 12/9.	**P_1, 2_**, **HCP:** Italian version of 40-item ICUESS; **P:** at ICU, day of transfer to ward; **PQ~**.
Carey et al., 2019, USA [47]	**SC:** 1; **P:** n = 16, -y, 12/4; cardiovascular ICU; coronary artery bypass graft surgery; no data on diagnoses.	**P:** 23-item-scale concerning stressful experiences, no name, adapted from Pennock (1994), during ICU stay, 12 to 24 h after surgery; **PQ−**.
Cochran & Ganong 1989, USA [3]	SC: 1; P: n = 20, 54 y, 10/10; no data on diagnoses; medical and surgical ICUs; HCP: n=23, -y, 3/20; 100% nurses.	**P, HCP:** 42-item ICUESS; **P:** 1–2 days after ICU; **HCP**: directly after ICU shift; **PQ~**.
Cornock 1998, UK [4]	**SC:** 2; **P:** n = 71, 59.0 y, -m/f; all patients ventilated, no data on diagnoses, 2 ICUs from 2 general hospitals; **HCP:** n = 71, 30 y, -m/f; 100% nurses.	**P, HCP:** 50-item ESQ; **P:** 2 days after ICU; **HCP**: during ICU shift; **PQ~**.
Desotte et al., 2016, Brazil [48]	**SC:** 1; **P:** n = 105, 58.8 y, 67/38; 2 ICUs, immediate postoperative cardiac surgery; coronary artery disease (55), mitral valve disease (42), coronary artery plus mitral valve disease (8).	**P:** 50-item adapted Brazilian ESQ, within 48 h after ICU, semi-structured interview; **PQ~**.
Dias et al., 2015, Brazil [34]	**SC:** 1; **P:** n = 60, various cardiological and surgical diagnoses; 2 subsamples in 2 ICUs, coronary ICU: n = 30, 55.6 y, 20/10; general postoperative ICU: n = 30, 53.6 y, 16/14.	**P:** 50-item adapted Brazilian version of ESQ, time of data collection and setting not specified; **PQ~**.
Granja et al., 2005, Portugal [5]	**SC:** 10; **P:** n = 464, 58.0 y ^+^, 281/183; 10 ICUs, no data on diagnoses, medical and surgical ICUs; admission modes: medical (46%), elective surgery (32%), non-elective (13%), multiple trauma (9%).	**P:** Recollection Questionnaire including 24 stressor items, 6 months after ICU discharge, at home, questionnaires sent by mail; **PQ−**.
Gültekin et al., 2018, Turkey [49]	**SC:** 1; **P:** n = 98, 55.1 y, 56/42; no data on diagnoses, general surgical and anesthesiological reanimation ICUs.	**P:** adapted Turkish 40-item ICUESS, ICU stay; PQ**−**.
Heidemann et al., 2011, Brazil [50]	**SC:** 1; **P:** n = 32, 56.5 y, 26/6; acute coronary syndrome (75%), congestive heart failure (9.3%), other cardiological diagnoses (9.3%); coronary ICU of a university hospital.	**P:** Brazilian 40-item version of the ICUESS, at days 1, 2, 3 after ICU admission; **PQ~**.
Hweidi 2007, Jordan, [51]	**SC:** 2; **P:** n = 165, 53.4 y, 97/68; no data on diagnoses; 3 ICUs in 2 governmental hospitals.	**P:** Jordanian version of the 42-item ICUESS, 2–3 days after discharge from ICU, structured interview, general wardm or intermediate unit; **PQ+**.
Hweidi & Nizamli 2015, Syria [52]	SC: 2; P: n = 150, 51.8 y, 96/54; no data on diagnoses; 2 ICUs in 2 governmental hospitals.	P: Arabic version of the 42-item ICUESS, 2 days after discharge from ICU, structured interview, general ward. PQ+.
Kalfon et al., 2010, France [6]	**SC:** 14; **P:** n = 868, 55.7 y, 538/330; no data on diagnoses; 14 surgical and medical ICUs in academic secondary and tertiary care centers.	**P:** 16-item IPREA, day of ICU discharge, structured interview; **PQ+.**
Locihova et al., 2018, Czech Republic [35]	**SC:** 1; **P:** n = 30, -y, 11/19; no data on diagnoses; admission modes: planned (63%), emergency (37%). **R:** n = 30, -y, 11/19; first degree relatives, at least 1 patient visit on ICU; **HCP:** n = 30, -y, 6/24; 100% nurses.	**P, R, HCP:** Czech version of 50-item ESQ; **P:** ICU stay, structured interview; **R, HCP:** same day as P interview, al ICU; **PQ~**.
Mistraletti et al., 2018, Italy [36]	**SC:** 1; **P:** n = 29, 63.0 y, 15/14; no data on diagnoses; general ICU, patients treated with awake/cooperative sedation; **R_1_:** n = 29, 47.0 y, 17/12; relatives of patient group; **R_2_:** n = 33, 51 y, 16/17; relatives of patients not included in study; **HCP:** n = 56, 33 y, 28/28; 16 nurses, 16 attending physicians, 24 students/trainees.	**P, R, HCP:** adapted Italian 33-item version of ICUESS; **P:** before discharge from ICU, structured interview; **PQ+.**
Nelson et al., 2001, USA [53]	**SC:** 1; **P:** n = 50, 63.0 y, 32/18; cancer patients at high risk for hospital death, medical ICU of a university-affiliated, tertiary-care, urban medical center.	**P:** modified ESAS, ratings of stress, pain and discomfort associated with ICU conditions and procedures; no data on item number; **PQ−**.
Novaes et al., 1999, Brazil [8]	**SC:** 1; **P:** n = 50, 52.8 y, 36/14; no data on diagnoses, general ICU; **R:** n = 50, -y, 11/39; first degree relatives, at least 1 patient visit on ICU; **HCP:** n = 50, -y, 18/32; 46% nurse attendants, 34% nurses, 10% physiotherapists, 8% physicians, 2% psychologists.	**P, R, HCP:** adapted Brazilian version of 40-item ICUESS; **P:** ICU stay, structured interview. **R, HCP:** same day as P interview, at ICU; **PQ−**.
Pagnucci et al., 2019, Italy [54]	**SC:** 1; **P:** n = 74, 68.5 y, 43/31; admission from surgical ward (47), medical ward (17), emergency department (10).	**P:** 33-item SEDAICU (based on ICUESS), at ICU, at the second day; **PQ+.**
Paldon et al., 2014, India [18]	**SC:** 1; **P:** n = 75, -y, 53/22; 44% cardiological diagnoses; 65% treated medically; medical, surgical, and cardiological ICUs. **HCP:** n = 75, -y, 26/49; 100% nurses.	**P, HCP:** 35-item stressor scale, no name, at ICU, day of transfer to ward; **HCP**: ICU shift; **PQ~**.
Pang & Suen 2008, 2009, Hong Kong, China, [37,42]	**SC:** 1; **P:** n = 60, 59.7 y, 44/16; 37 with emergency admission, 23 after elective surgery; no data on diagnoses; 1 ICU of a regional hospital; **R:** n = 60, 41.9 y; 22/38; ‘interacting individuals related by blood, marriage or adoption’, at least 1 patient visit on ICU **HCP:** n = 54, -y, 11/43; 100% nurses.	**P, R, HCP:** Chinese version of 50-item ESQ, structured interview; **P:** at ICU, time not specified; **R, HCP:** time and setting not specified; **PQ+**.
Patacky et al., 1985, USA [55]	**SC:** 1; **P:** n = 27; -y, 17/10; patients with myocardial infarction, coronary ICU of a university hospital.	**P:** 20-item CCSM, at ICU, during stay at coronary care unit; structured interview; **PQ−**.
Pennock et al., 1994, USA [7]	**SC:** 1; **P:** n = 127, 63.9 y, 91/36; after coronary artery bypass craft surgery; surgical ICU.	**P:** 25-item stressor scale, no name, 2 days after ICU stay, at thoracic unit, structured interview; **PQ−**.
Pieris et al., 2018, Sri Lanka [19]	**SC:** 32; **P:** n = 438, 45.0 y ^+^, 207/231; various diagnoses; 32 state ICUs: 19 mixed general, 6 medical, 7 surgical.	**P:** semi-structured telephone interview, adapted version of Recollection Questionnaire including 23 stressor items, 30 days after ICU discharge; **PQ−**.
Rattray et al., 2010, United Kingdom [12]	**SC:** 6; **P:** n = 103, 60.0 y, 65/37 ^++^; no data on diagnoses, all mechanically ventilated; 6 ICUs from 6 hospitals.	**P:** 31-item ICEQ, on ward, at hospital discharge, structured interview, only data on 24/31 items are reported; **PQ+**.
Rosa et al., 2008, Brazil [56]	**SC:** 2; **P:** n = 106, 57.4 y, 60/46; various diagnoses; two ICUs, in a public and a private hospital.	**P:** 50-item Brazilian version of ESQ, structured interview, during hospital stay, time and setting not specified; **PQ+**.
Rotondi et al., 2002, USA [1]	**SC:** 1; **P:** n = 100, 53.2 y, 47/53; no data on diagnoses; all received mechanical ventilation for at least 48 h. Four ICUs from a tertiary-care university medical center.	**P:** ICU-SEQ, 10 items ETT experience, 22 items ICU experience; structured interview, after discharge from ICU, at ward; **PQ~**.
Rowe & Weinert 1987, USA [57]	**SC:** 3; **P:** n = 78, -y, 49/21 ^++^; 22 myocardial infarction, 41 unspecified non-MI diagnoses, 15 unknown diagnosis; 3 cardiac care units of 3 hospitals.	**P:** 30-item HESS, during stay at CCU; **PQ+**.
Sharmila et al., 2016, India [38]	**SC:** ?; **P:** n = 40, -y, 26/14; ICUs of selected hospitals; no data on numbers; cardiological conditions in 27.5% patients, no further data on diagnoses; **HCP:** n = 40, -y, 3/37; 100% nurses.	**P, HCP:** adapted Indian version of 50-item ESQ, **P:** ICU stay; **HCP**: setting and time not specified; **PQ~**.
So & Chan 2004, Hong Kong [39]	**SC:** 2; **P:** n = 50, 59.4 y, 36/14; 3 ICUs of 2 hospitals; admissions: 92% urgent, 8% elective surgical; no data on diagnoses. **HCP:** n = 92, -y, 11/81, 100% nurses (82 registered nurses, 10 nursing officers, ward managers or nurse specialists).	**P, HCP:** Chinese version of the 42-item ICUESS, **P:** 48 h after discharge from ICU, structured interview. **HCP**: ICU shift, directly involved in care of P sample; **PQ+**.
Soehren 1995, USA [24]	**SC:** 1; **P:** n = 43, 62.0 y, 34/9; surgery type: 34 bypass, 3 valve, 3 both; no data on diagnoses; cardiac ICU from tertiary care hospital.	**P:** 40-item ICUESS, at telemetry unit, 1–3 days after discharge from ICU; **PQ+**.
Soh et al., 2008, Malaysia [58]	**SC:** 2; **P:** n = 70, 46.4 y, 47/23; all ventilated; no data on diagnoses; 5 ICUs of 2 tertiary hospitals; general, urology, coronary.	**P:** Malaysian version of the 50-item ESQ, after transfer from ICU to general ward, structured interview; **PQ−.**
Soh et al., 2014, Malaysia [59]	**SC:** 4; **P:** n = 68 of 104 ^+++^; all ventilated; no data on diagnoses; ICUs of 4 governmental hospitals; 36 of 104 patients were not able to remember experiences of ICU stay.	**P:** Recollection Questionnaire including 24 stressor items, at ward, at least 3 days after ICU discharge; **PQ−.**
Takashima et al., 2018, Japan [60]	**SC:** 1; **P:** n = 96, 69.4 y, 74/22; all mechanically ventilated for >12 h; general ICU; treatment at admission: 64 cardiovascular surgery, 18 digestive surgery, 14 other.	**P:** extended Japanese version of ICU-SEQ, 10 items ETT experience, 24 items ICU experience; structured interview, during discharge from ICU, at ICU; **PQ~.**
Turner et al., 1990, South Africa [61]	**SC:** 1; **P:** n = 100, 38.1, 45/55; diverse diagnoses, 68 mechanically ventilated; respiratory ICU.	**P:** No information on stressor measure available, at ward, within 48 h of ICU discharge; structured interview; **PQ−.**
Wilson 1987, USA [62]	**SC:** 1; **P:** n = 32, 51.0 y, 18/14; no data on diagnoses; surgical ICU.	**P:** 22-item Patient Stressor Scale, at ward, at least 48 h after transfer from ICU; structured interview; age over 14 years (mean age 51 years); **PQ+**.
Yava et al., 2011, Turkey [40]	**SC:** 2; **P:** n = 155, 55.6 y, 107/48; 53 medical and 102 surgical ICUs; 68 planned, 87 unplanned or emergency. Various reasons for hospitalization. **HCP:** n = 152, 30.32 y, -m/f; 100% nurses, all working at SC ICUs of university hospitals.	**P, HCP:** Turkish version of 50-item ESQ ^(a)^, **P:** at ward, within 2 days after ICU stay, semi-structured interview; **PQ+**.
Zengin et al., 2020, Turkey [63]	**SC:** 1; **P:** n = 116, 57.8 y, 60/56; no data on diagnoses; general ICU of university hospital.	**P:** 14-item questionnaire, no name, on ICU, directly before transfer to other wards; structured interview; **PQ+**.

^(a)^ authors report to have applied the ICUESS comprising 50 items; however, they list the 50 items of ESQ; +: only data on median age available; ++ gender data missing for 1 patient in Rattray et al. (2010), for 8 patients in Rowe & Weinert (1987); +++ data on age and gender missing for the relevant subgroup of 68 patients in Soh et al. (2014). Abbreviations: **CCSM:** Coronary Care Stress Measurement; **CCU:** cardiac/coronary care unit; **ESAS:** Edmonton Symptom Assessment Scale; **ESQ**: Environmental Stressors Questionnaire, an adaptation and extension of the ICUESS; ESQ for relatives and HCPs with the instruction to rate patients’ stressors from their perspective; **ETT:** endotracheal tube; **HCP**: Health care provider; **HESS:** Hospital Events Stress Scale; **ICEQ:** Intensive Care Experience Questionnaire; **ICU:** Intensive care unit; **ICUESS:** Intensive Care Unit Environmental Stressor Scale; ICUESS for relatives and HCPs with the instruction to rate patients’ stressors from their perspectives; **ICU-SEQ:** ICU Stressful Experience Questionnaire; **IPREA**: Inconforts des Patients de REAnimation; **m/f:** ratio of men to women; **-m/f:** data on gender distribution missing; **n.s.:** not significant; **P**: Patient; **PQ+/−:** data on psychometric quality of stressor measure available/not available; **PQ~:** some data can be interpreted as aspects of construct validity; **R**: Relatives; **SC:** study center; **SEDAICU:** Stress Factors in Intensive Care Unit Questionnaire; **y:** year; **-y**: data on average age missing.

### 3.2. Data on Perceived Stressors in the ICU

As shown in Table 1, 52.4% of the studies applied either the ICUESS (n = 11 [26.2%]) or the ESQ (n = 11 [26.2%]) to measure the perceived severity of stressors. The Recollection Questionnaire, the ICEQ, and the ICU-SEQ were used in three (7.1%), two (4.8%), and two (4.8%) investigations, respectively. The remaining 13 studies used instruments that were not applied in any one of the other included studies. None of the articles reported results of comprehensive psychometric analyses of the stressor questionnaires. However, 15 studies provided selected psychometric data on reliability and validity (35.7%), and another 15 studies (35.7%) reported data suggesting diverse aspects of construct validity (Table 1). The Supplementary Table S1 comprises all extracted stressor items, sorted by studies, and within studies sorted by patient, HCP, and relative samples. There are 45 ranking lists from patient samples with a median of 10 items (min: 5, max: 21; IQR: 10–12) and 14 ranking lists from HCPs samples, also with a median of 10 items (min: 4, max: 26; IQR: 10–11.5). Finally, 5 ranking lists from relative samples are available, with 10 items in 4 samples, and 18 items in 1 study.

Including duplicate items, we extracted 491 stressors in patient samples, 156 stressors in HCP samples, and 58 stressors in samples of relatives (Supplementary Table S1). Since all stressors rated by HCPs and relatives, respectively, were also rated by patients, further analyses were only carried out for the 491 stressors of ranking lists from the patient samples. After removal of duplicates and overly redundant items, the stressor items were grouped into domains. In total, our final stressor item set consisted of 137 stressors: 46 items of the domain *‘Physical, treatment, and disease-related stressors’*, 55 items of the domain *‘Mental Health’*, 21 items of the domain *‘Communication’*, and 15 items of the domain *‘Environment’* (Table 2, and Supplementary Table S2). Concerning the agreement among the Q sortings of the raters, the ICCs were 0.81 for *‘Physical, treatment, and disease-related stressors’*, 0.76 for *‘Mental Health’*, 0.77 for *‘Communication’*, and 0.61 for *‘Environment’*. According to Cicchetti & Sparrow 1981, the quality of inter-rater reliability is indicated by the size of ICCs, with lower than 0.40: poor; between 0.40 and 0.59: fair; between 0.60 und 0.74: good; and equal or higher than 0.75: excellent [64]. Thus, the inter-rater reliability of the four Q-sort raters was good for the stressor severity rankings of ‘Environment’ and excellent for the three other domains. Table 2 shows the stressors after they were Q-sorted. The most severe stressor per domain is allocated to the rank ‘1′, and the least severe stressor by the last rank, e.g., rank 1 ‘Fear of death’, and rank 41 ‘Being bored’ in the list of mental health stressors. Thus, smaller ranking scores indicate higher levels of stress severity. The numbers of severity ranks depend on the number of stressors, and because of the shared ranks, the number of ranks can be smaller than the number of items, e.g., 46 items allocated to 38 ranks and in the physical stressor list (Table 2).

**Figure 2:**
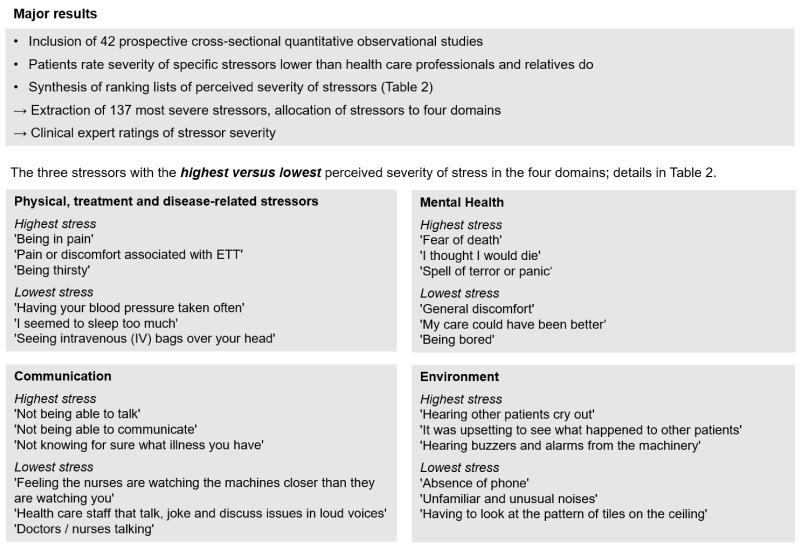
Summary of the results of the systematic review.

There were two major results regarding the comparisons of stressor rankings of patients, HCPs, and relatives. First, the stressor ranking lists of the three groups consisted of rather similar items, and the lists of HCPs and relatives did not contain any stressors that were not included in the lists of the patients (Supplementary Table S1). Second, data indicate that the patients rate the severity of specific stressors lower than HCPs and relatives do. In total, there were 13 studies with 14 comparisons of general stressor severity ratings of patients and HCPs (Supplementary Table S1). Ten of these fourteen comparisons showed statistically significant lower perceived stressor intensity in patients than in HCPs [3,23,33,35,36] (two comparisons); [8,18,37,40,42]. One study found no statistically significant differences between patients and HCPs [41], and three studies lack information on the statistical significance test parameters [4,38,39]. Descriptive data of two of these studies suggest lower stressor intensity ratings of patients [4,39], and data of one study suggest higher stressor intensity ratings of patients [38]. Four studies provided data on five comparisons of patients and relatives. Out of the five comparisons, four showed statistically significant lower stressor intensity ratings in patients [35,36] (two comparisons), [42], and one study found no significant difference between patients and relatives [8]. Figure 2 summarizes the main results of the systematic review and semi-quantitative analysis.

**Table 2:** Synthesis of perceived severity of stressors in the ICU: Ranking lists of four stressor domains according to mean scores of severity rankings of four clinical ICU experts ^+^.

	**Mean Rank**		**Mean Rank**
**Physical, treatment and disease-related stressors (46 items)**			
**1.** Being in pain	1.25	**21.** Daily needle punctures	18.75
**2.** Pain or discomfort associated with ETT	3.00	**22.** Having the sensation of not eating	19.00
**3.** Being thirsty	3.75	**23.** Having to be assisted with the bedpan	19.50
**4.** Shortness of breath	6.00	**24.** Bed-related discomfort	19.75
**5.** Pain and discomfort anticipated as the results of treatment and unexplained procedures	6.50	**24**. Being bedridden	19.75
**6.** Machine (ventilator) dependence	7.75	**24**. Emergency measures being performed	19.75
**7.** Having tubes in your nose and/or mouth	8.25	**25.** Chest tube removal	20.00
**8.** Frequent interruptions of sleep	8.75	**26.** Not being able to eat	20.50
**9.** Not being able to sleep	9.00	**27.** Having major surgery	20.75
**10.** Sleeplessness	9.25	**28.** Being admitted to the ICU	22.25
**11.** Difficulties in sleeping	12.00	**28.** Difficulties swallowing	22.25
**12.** Coughing while receiving mechanical ventilation	12.75	**29.** Sore throat	23.00
**13.** Sleep deprivation	13.50	**30.** Being unable to move freely in bed because of equipment	23.50
**14.** Endotracheal tube aspiration/suctioning (ETT)	14.00	**31.** Frequent physical exams by doctors and nurses	24.25
**14.** Secretion	14.00	**31.** Not being able to go to the bathroom	24.25
**15.** Feeling of cold	14.25	**32.** Having to wear an oxygen mask	25.75
**16.** Not being able to move as you wish	14.50	**32.** Urinary catheter	25.75
**16.** Not being able to move freely	14.50	**33.** Hearing your heart monitor alarm go off	26.00
**17.** Hunger	15.00	**34.** Being awakened by nurses	28.25
**18.** Feeling of heat	16.50	**35.** Frequent drawing of blood	29.25
**19.** Being restricted by tubes/lines	17.25	**36.** Having your blood pressure taken often	30.50
**19.** Having a sudden hospitalization you weren’t planning to have	17.25	**37.** I seemed to sleep too much	34.25
**20.** Being stuck with needles	18.50	**38.** Seeing intravenous (IV) bags over your head	35.00
	**Mean Rank**		**Mean Rank**
**Mental health stressors (55 items)**			
**1.** Fear of death	1.00	**23.** Loneliness	16.50
**2.** I thought I would die	1.50	**24.** Insecurity	17.25
**3.** Spell of terror or panic	2.25	**25**. Depression	17.50
**4.** Agony/panic	3.75	**26.** Frustration	17.75
**4.** Anxiety/fear	3.75	**27.** Isolation	18.50
**5.** Being worried/scared	5.00	**28.** Fear of AIDS	19.25
**6.** Feeling something bad will happen	5.25	**29.** Not knowing the length of stay in ICU	19.75
**7.** Knowing that you have a serious illness	8.50	**30.** Only seeing family and friends for a few minutes each day	20.75
**8.** Thinking you might lose your sight	8.75	**30.** I was constantly disturbed	20.75
**9.** Nightmares	9.25	**31.** Having no privacy	21.25
**10.** Fear of medical procedures	10.25	**31.** Concerns about their health and its impact on their daily life	21.25
**11.** I had bad dreams	10.50	**32.** Not knowing what day it is	21.50
**12.** I felt scared	10.75	**33.** Financial worries	21.75
**13.** I felt helpless	12.00	**33.** Limits on visiting by your family and friends	21.75
**13.** Not having control over their activities	12.00	**34.** Losing track of time	22.00
**14.** My memories in intensive care are frightening	12.25	**35.** I wish I remembered more about it	23.50
**15.** Uncertainty about the future	13.25	**36.** Not knowing what time it is	24.00
**16.** Thinking you might be having a heart attack	13.50	**36.** I felt tense	24.00
**17.** Not being in control of yourself	14.25	**36.** I wish I had known more about what was happening to me	24.00
**18.** Confusion	14.75	**37.** Losing contact with the outside world, dates, and time of day	24.50
**19**. I saw strange things	15.25	**37.** Being unable to fulfil family roles	24.50
**19.** Family worries	15.25	**37.** Not being able to fulfil family responsibilities	24.50
**20.** Separation from family	15.50	**38.** Most of my memories of intensive care were blurred	26.00
**20.** Missing your spouse	15.50	**38.** Fear of hospital-transmitted diseases	26.00
**20.** Missing your loved ones/family and friends	15.50	**39.** General discomfort	27.00
**21.** Concern for family	15.75	**40.** My care could have been better	29.00
**22.** Not knowing where you are	16.00	**41.** Being bored	30.00
**22.** Feeling anxiety about ETT	16.00		
	**Mean Rank**		**Mean Rank**
**Communication stressors (21 items)**			
**1.** Not being able to talk	2.25	**9.** Not knowing when to expect things to be done	7.50
**1.** Not being able to communicate	2.25	**10.** Being pressurized to consent to treatment	8.25
**2.** Not knowing for sure what illness you have	4.25	**11.** Having doctors and nurses talk about you rather than to you	9.50
**3.** Not being told what your diagnosis is	4.75	**11.** Having the nurse/HCPs be in too much of a hurry	9.50
**4.** Not having treatments explained to you	5.00	**12.** Having nurses and doctors talk too fast or use words you can’t understand	10.25
**4.** Not knowing or understanding your illness and its seriousness	5.00	**13.** Hearing people talk about you	11.00
**4.** Communication difficulties	5.00	**14.** Being examined by several different doctors and nurses who ask the same questions	11.50
**5.** Lack of information	6.00	**15.** Feeling the nurses are watching the machines closer than they are watching you	12.75
**6.** Not knowing when to expect things to be done to you	6.50	**16.** Health care staff that talk, joke, and discuss issues in loud voices	13.00
**7.** Not knowing the results or reasons for your treatment	6.75	**17.** Doctors/nurses talking	14.50
**8.** Dependence on doctors and nurses	7.25		
	**Mean Rank**		**Mean Rank**
**Environmental stressors (n=15)**			
**1.** Hearing other patients cry out	2.00	**9.** The noise around you	6.50
**2.** It was upsetting to see what happened to other patients	2.50	**9.** Limited visiting hours	6.50
**3.** Hearing buzzers and alarms from the machinery	4.00	**10.** Uncomfortable bed or pillow	6.75
**4.** Alarms	4.25	**11.** Being in a room which is too hot or cold	8.00
**5.** Excessive light	4.50	**12.** Absence of phone	9.00
**6.** Noise from machines	5.50	**13.** Unfamiliar and unusual noises	9.50
**7.** Watching treatments being given to other patients	6.00	**14.** Having to look at the pattern of tiles on the ceiling	10.50
**8.** Having lights on constantly	6.25		

+ Rankings were performed on 137 substantial stressors according to the perspective of patients. ***Higher*** stress severity is indicated by ***smaller mean ranking scores***. Ranges of severity ranks depend on the number of stressors. However, because of shared ranks, the number of ranks does not equal the number of stressor items. Ranks reach from 1 (most severe stressor) to 38, 41, 17, and 14 (least severe stressor) for the domains of *(1) Physical, treatment, and disease-related stressors (46 stressor items), (2) Mental health (55 stressor items), (3) Communication (21 stressor items), and (4) Environment (15 stressor items)*, respectively. Severity ranks were produced by four clinical ICU experts applying an adapted version of Q sort technique of [31,32]; data source: 42 original studies from 1981 to 2020 (details and major results of the included studies see Table 1 and Supplementary Table S1).

## 4. Discussion

In this systematic literature review, we synthesized the results of 42 prospective cross-sectional quantitative observational studies that had identified severity rankings of perceived stressors in ICUs. Out of all ranking lists, we extracted 137 stressor items that were most frequently ranked among the most severe stressors. After allocation to four domains, a group of clinical ICU experts sorted the extracted items with good to excellent agreement according to their stress levels. The results of studies comparing stressor rankings of patients, HCPs, and relatives suggest that patients rate the severity of specific stressors lower than HCPs and relatives do.

Study characteristics showed that the investigations were performed in diverse international regions, with a median patient sample size of 76.5. Gender distribution and age were similar to those of other ICU studies that had applied questionnaires or interviews to investigate the experience of ICU patients (e.g., [9,10,28,65,66,67,68,69,70,71,72,73,74,75]): With an average rate of 64%, most of the patients were men and had a median age of 55.7 years. There were 13 studies that had also included stressor ratings of HCPs who were mostly women (68.4%) and were between 30 and 40 years of age. Importantly, with a median rate of 100%, the majority of studies involved exclusively nurses and neglected the perspectives of other HCPs such as physicians, respiratory therapists, and psychologists. In the four studies involving relatives, there were higher ratios of women that ranged from 41% to 78%, and an average age between 42 and 51 years, which is moderately younger than the age of patients.

### 4.1. Implications for Clinical Research and Practice

The findings of this systematic review can contribute to reducing stressful experiences of ICU patients by improving the way how HCPs and relatives understand the patients’ perceptions of ICU stressors. The extracted stressors may serve as an item base to inform the construction of new stressor assessment instruments including innovative communication tools [73,76]. For this purpose, five aspects should be taken into account. (1) HCPs and relatives seem to concordantly overestimate the general stressor intensities. However, there are also research findings that showed that HCPs sometimes underestimate negative and overestimate positive experiences of patients. In a study of Schindler et al. (2013), for example, HCPs underestimated patients’ stressful experiences of fatigue and worries concerning the future, but they overestimated the extent of patients feeling treated kindly by the staff and having trust in them [75]. (2) While all high-ranking stressors that were identified by HCPs and relatives were also mentioned by patients, we have to keep in mind that the 491 stressors of the patient rankings included many experiences that HCPs (156 items) and relatives (58 items) did not rate as highly stressful (details see Supplementary Table S1). Among these stressors, which are included in the final list of 137 stressors, are ‘Shortness of breath’, ‘Frequent interruptions of sleep’, ‘Nightmares’, ‘Bad dreams’, ‘Feeling helpless’, ‘Depression’, ‘Not being told what your diagnosis is’, ‘Dependence on doctors and nurses’, ‘Having doctors and nurses talk about you rather than to you’, and ‘It was upsetting to see what happened to other patients’. (3) The majority of the reviewed ICU stressor questionnaires are either older than 20 years or cultural adaptations of the ICUESS and the ESQ. An inspection of the 137 stressor items in Table 2 reveals that some stressor items might be reformulated with contemporary wordings to ensure the clinical utility of new item lists. (4) This work covers studies published between 1981 and 2020. During these four decades, ICU standards have improved, and chances of survival after critical illness have dramatically increased [14,77,78]. The question may arise as to what extent these changes might have influenced how patients experience ICU stressors. An analysis of potential changes of perceived severity of ICU stressors over time is beyond the scope of the present review and should be performed as a major study objective in a future review. A first inspection of studies from different decades may give preliminary insights that can inform further detailed analyses of this topic. Three studies from different decades enable a first comparison of stressor ranking lists: the pioneering study of Cochran & Ganong (1989) [3], the study of Biancofiore et al. (2005), which had been published 16 years later [33], and the most recent study of Zengin et al. (2020) [63] (for details, see Supplementary Table S1). On the one hand, each ranking list contains stressors among the 10 highest ranking items that are not included in the other lists. On the other hand, the lists of all three studies contain the major stressors of pain, thirst, tubes, difficulties in sleeping, and missing loved ones. A tentative interpretation would assume that there are core stressors that are perceived as highly stressful, and that this experience is less dependent on changes in ICU standards over time. Sophisticated analyses will be necessary to find out which specific stressors have been rated differently over the last four decades of improvement of critical care. (5) Many of the mental and physical health stressor items refer to different aspects of relatively similar experiences. As a consequence, the numbers of stressor items in the mental and physical health domains are substantially larger than the ones in the domains of communication and environment. The larger item number of these domains is also a result of the finding that the ranking lists of patients, HCPs and relatives contain more physical and mental health stressors than communication and environmental stressors (Table 2). However, we have to take into consideration that some studies showed moderate to large associations between perceived environmental and communication stressors on the one hand and mental distress on the other hand, even when the stress level of the environmental and communication stressors were relatively low [10,63]. Moreover, there is ample evidence that perceived communication stressors and objectively measured environmental stressors can cause subjective mental distress, and that a reduction of environmental and communicative stress level leads to an improvement in mental distress [11,50,54,65,67,71,72,79,80]. Thus, it is very important to ask also for these stressors, although they seem to be less prominent and perceivable for patients, as compared to physical and mental health stressors. By doing so, we might detect environmental and communication disturbances that cause mental and physical stress without patients being able to identify these associations by themselves. When designing novel tools such as stressor assessment instruments, researchers may select items from all four domains that are not overly redundant and of different extent of stress levels. The subscales should contain a minimum of 5 items to ensure sufficient internal consistency, and they should not exceed a maximum of approximately 10 items to avoid the risk of lengthy questionnaires overcharging the limited resources of critically ill patients.

### 4.2. Limitations and Future Directions

There are essential patient ICU experiences that were not included in this review. In order to rate the intensity of perceived stressors, patients need to be able to remember their experiences. However, findings showed that patients sometimes have memory problems concerning their ICU treatment. Several studies found indeed that between 15% and 38% of ICU patients did not remember their experiences on ICU, and approximately 50% had difficulties to distinguish dreams and reality [1,5,9,12,27,28,59,60,81]. This means that stress perception of this considerably large group of ICU patients has either been distorted or has not been included in the reviewed questionnaire investigations. Moreover, our focus on stressful experiences bears the risk to neglect patients’ reports of positive experiences and resources during treatment of their critical illness. With 59% to 94%, the majority of patients link the ICU treatment of critical illness with positive experiences, such as feeling in good hands, in a friendly and calm environment, and well cared for and supported by HCPs and family [9,12,19,75,82]. While Wassenar et al. (2014) provided the first qualitative systematic review on factors contributing to ICU patients’ feeling of safety [80], meta-analyses on recollections and positive experiences of ICU patients are lacking.

Limitations are also due to methodological restrictions. The literature search for this review ended in May 2020, and we did not include articles that were published afterwards. In a rerun of the search on 19 August 2021, we found one new article that fulfilled the inclusion criteria [83]. All of the 10 most severe stressors found by Coelho et al. (2021) [83] were among the 137 stressor items extracted in our analyses, suggesting that the results of the review are rather robust. Especially the stressor experience of the relatives may be quite dependent on different cultural contexts. However, the study selection was limited to articles written in English, and 83 % of the papers came from North America, Europe, and Asia. Thus, we should keep in mind that several regions of the world are underrepresented in this review, and that potentially relevant articles have been excluded because of methodologically strict language limitations. Furthermore, the literature review is restricted to quantitative studies. Yet, several recent qualitative and mixed methods studies have been conducted that contribute substantially to a deeper understanding of how patients, their relatives, and HCPs experience treatment in an ICU, including ICU stressors (e.g., [9,27,28,29,84,85,86,87,88,89,90,91,92,93]). These studies address to a greater extent patient preferences, the stressful experiences of relatives, as well as innovative concepts to reduce ICU stress. They also explore the associations of stressful experiences with symptoms and consequences of post-intensive care syndrome (PICS), as well as mental distress, primarily delirium, depression, anxiety, and trauma-related disorders. Finally, our analyses have been performed with descriptive and semi-quantitative methods. Indeed, we decided to conduct the analyses without the application of classical quantitative methods of meta-analyses in order to include a broad range of relatively heterogeneous studies.

Taken together, future systematic reviews on experiences of ICU patients might synthesize data on the rates of ICU patients who have no recollection or problems remembering experiences on ICU. Furthermore, focussing explicitly on positive experiences and resources on the ICU rather than stressful experiences would be another important objective to investigate. The inclusion of studies that are not published in English would contribute to higher cultural diversity of future reviews. Meta-analyses of perceived stressors on ICU would be desirable. However, they could only be meaningfully performed after having resolved the issue of relatively heterogeneous item formulations and translations of the contemporary questionnaires of perceived stressors.

## 5. Conclusions

In this systematic literature review, we extracted 137 stressors of patients treated in the ICU. Patients, relatives, and HCPs rated the perceived severity of these stressors as substantially high. We allocated the stressors to four domains, sorted by clinical ICU experts according to stress levels. These ranking lists may serve to improve HCPs’ and relatives’ understanding of patients’ perception of stressors by using them in the development of new assessment instruments of stressors in the ICU, including novel IT-based communication tools.

## Data Availability

The data presented in this study are available on request from the corresponding author.

## Supplementary Materials

The following are available online at https://www.mdpi.com/article/10.3390/jcm10173928/s1, Table S1: Most severe stressors of patients treated in the ICU—Summary of the results of 42 studies on rankings of perceived stressors, Table S2: Synthesis of most severe perceived stressors in the ICU, patient ratings, sorted in alphabetic order; source: 42 original studies from 1981 to 2020.
