# Supplementary material for: Perceived Severity of Stressors in the Intensive Care Unit: A Systematic Review and Semi-Quantitative Analysis of the Literature on the Perspectives of Patients, Health Care Providers and Relatives
Source: J Clin Med. 2021 Aug 31;10(17):3928. doi: 10.3390/jcm10173928 (PMC8432195; doi:10.3390/jcm10173928)
Supplement: Supplementary file 1 [file jcm-10-03928-s001.zip › jcm-1347311-supplementary.pdf]

Supplementary Table S1: Most severe stressors of patients treated in the ICU - Summary of the results of 42 studies on rankings of perceived stressors *(abbreviations see caption).*

**Author, Year**<br>**Country**	**Main Results:**<br>**- Comparison of general stressor rating intensities of Ps, HCPs and Rs**<br>**- Ranking list of strongest stressors, starting with rank 1; up to ten ranks [a)]**
Abuatiq 2015 [23]	**General stress ratings, mean total ESQ scores:** P < HCP; p<0.001<br>**Strongest stressors, % agreement:**<br>**P:** 'being in pain', 'not being able to sleep', 'financial worries', 'being restricted by tubes/lines'-'being unable to fulfil family roles' [2 items sharing rank 4], 'not being in control of yourself', 'having tubes in your nose or mouth'-'not knowing the length of stay in ICU' [2 items haring rank 6], 'not being able to move your hands or arms because of intravenous (IV) lines'-'being stuck with needles' [2 items sharing rank 7], 'missing your husband or wife'-'not knowing when to expect things to be done'-'not being able to communicate' [3 items sharing rank 8], 'being in a room which is too hot or cold', 'being thirsty'.<br>**HCP:** 'being in pain', 'not being able to communicate', 'not being in control of yourself', 'financial worries'.
Alasad et al 2015 [43]	**Strongest stressful experiences, % agreement:**<br>**P, % agreement items:**<br>'I wish I had known more about what was happening to me', 'I seemed to sleep to much', 'It was upsetting to see what happened to other patients', 'I never knew whether it was day or night', 'my memories in intensive care are frightening', 'It was always too noisy', 'most of my memories of intensive care were blurred' - 'I was constantly disturbed' [2 items sharing rank 7], 'I thought I would die', 'my care could have been better'.<br>**P, % agree to frequency items, all/most of the times:** 'I seemed to be in pain', 'I felt helpless', 'I felt scared', 'I had bad dreams', 'I saw strange things'.
Azizi-Fini et al 2017 [41]	**General stress ratings:** no difference between P and HCP, p>0.05<br>**Strongest stressors, mean item scores:**

Author, Year Country	Main Results: - Comparison of general stressor rating intensities of Ps, HCPs and Rs - Ranking list of strongest stressors, starting with rank 1; up to ten ranks [a)]
	**P:** 'not being in control over urine and stool', 'being in pain', 'having tubes in your nose or mouth', 'not being able to communicate', 'missing your family', 'fatigue', 'not being able to sleep', 'fear of death', 'being unable to fulfil family roles', 'not knowing the length of stay in ICU'. **HCP:** 'not being in control over urine and stool', 'fear of death', 'being in pain', 'having tubes in your nose or mouth', 'being restricted by tubes/lines', 'not being able to communicate', 'fatigue', 'missing your family', 'not being able to sleep', 'hearing your heart monitor alarm go off'.
Ballard 1981 [2]	**Strongest stressors, P, rank sorting order:** 'being tied down by tubes', 'being in pain', 'being thirsty', 'missing your spouse', 'having to wear an oxygen mask', 'not being able to move your hands or arms because of intravenous lines', 'having to take breathing treatments', 'only seeing family and friends for a few minutes each day', 'being awakened by nurses', 'not knowing when to expect things will be done to you'.
Bergbom-Engbert & Haljamae 1989 [44]	**Strongest stressful experiences, P, most frequently reported:** 'anxiety / fear', 'not able to talk', 'secretion', 'pain', 'difficulties in sleeping', 'agony/panic', 'suctioning', 'insecurity', 'nightmares', 'extubation / decannulation'.
Bernal Ruiz et al 2018 [45, 46]	**Strongest stressors, mean item scores, P:** 'concerns about my health and its impact on my daily life', 'being unable to go to the bathroom', 'being unable to fulfil my family role', 'not being in control of my activities', 'being in pain', 'not being able to move your body parts because of being connected to cables, tubes, lines, or IV catheters', 'missing family or friends', 'not being able to sleep', 'often hearing unfamiliar and unusual noises from the machines around you', 'being stuck with needles'.
Biancofiore et al 2005 [33]	**General stress ratings**: $P_2 < P_1 < HCP_2 < HCP_1$, $p<0.001$ **Strongest stressors, mean item scores:** **P_1**: 'not being able to sleep', being in pain', 'having tubes in your nose or mouth', 'missing your husband or wife', 'only seeing family and friends for a few minutes each day', 'being tied down by tubes', 'being thirsty', 'hearing your heart monitor alarm go off', 'not being in control of yourself', 'uncomfortable bed or pillow'.

Author, Year<br>Country	Main Results:<br>- Comparison of general stressor rating intensities of Ps, HCPs and Rs<br>- Ranking list of strongest stressors, starting with rank 1; up to ten ranks [a)]
	**P_2**: 'only seeing family and friends for a few minutes each day', 'not being able to sleep', 'being in pain', 'having tubes in your nose or mouth', 'missing your husband or wife', 'being thirsty', 'being tied down by tubes', 'not being in control of yourself', 'having lights on constantly', 'uncomfortable bed or pillow'.<br>**HCP_1:** 'having tubes in your nose and/or mouth', 'being in pain', 'having no privacy', 'not having treatments explained to you', 'not being in control of yourself', 'not being able to sleep', 'only seeing family and friends for a few minutes each day', 'being tied down by tubes', 'missing your husband or wife', 'hearing other patients cry out'.<br>**HCP_2:** 'being in pain', 'having tubes in your nose and/or mouth', 'not being able to sleep', 'being thirsty', 'not being in control of yourself', 'being tied down by tubes', 'hearing other patients cry out', 'hearing your heart monitor alarm go off', 'missing your husband or wife', 'having no privacy'.
Carey et al 2019 [47]	**Strongest stressful experiences, mean item scores, P:** 'pain', 'endotracheal tube', 'sleeplessness', 'inability to talk', 'coughing while receiving mechanical ventilation', 'being confined to bed', 'endotracheal tube pain', 'not seeing family', 'doctors/nurses talking', 'concern for family'.
Cochran & Ganong 1989 [3]	**General stress ratings:** P < HCP, p<0.01 to p<0.0001<br>**Strongest stressors, mean item scores:**<br>**P:** 'having tubes in your nose and/or mouth', 'being stuck with needles', 'being in pain', 'not being able to sleep', 'being thirsty', 'not being in control of yourself', 'being tied down by tubes', 'missing your husband or wife', 'having to wear oxygen'-'only seeing family and friends for a few minutes each day'-'not knowing what day it is' [3 items sharing rank 9], 'not knowing where you are'.<br>**HCP:** 'being in pain', 'not being in control of yourself', 'having tubes in your nose or mouth'-'being tied down by tubes' [2 items sharing rank 3], 'not having treatments explained to you'-'not being able to sleep'-'being stuck with needles' [3 items sharing rank 4], 'hearing the buzzers and alarms from the machinery'- 'not knowing where you are' [2 items sharing rank 5], 'hearing your heart monitor alarm go off', 'missing your husband or wife', 'having strange machines around you', 'being thirsty'-'hearing other patients cry out' [2 items sharing rank 9], 'having the nurses be in too much of a hurry'-'having lights on constantly' [2 items sharing rank 10].
Cornock 1998 [4]	**General stress ratings:** P < HCP; no data on statistical significance

Author, Year<br>Country	**Main Results:**<br>**- Comparison of general stressor rating intensities of Ps, HCPs and Rs**<br>**- Ranking list of strongest stressors, starting with rank 1; up to ten ranks [a)]**
	**Strongest stressors, rank order, highest frequency out of 'list of 3 most stressful items':**<br>**P:** 'having tubes in your nose or mouth', 'being thirsty', 'not knowing what time it is', 'uncomfortable bed or pillow', 'being restricted by tubes/lines'-'being in pain'-'fear of death' [3 items sharing rank 5], 'not being able to sleep', 'not being able to communicate' [2 items sharing rank 6], 'having to wear oxygen', 'feeling the nurses are watching the machines closer than they are watching you', 'only seeing family and friends for a few minutes each day', 'missing your husband or wife'-'hearing people talk about you' [2 items sharing rank 10].<br>**HCP:** 'being in pain', 'not being able to communicate', 'not being in control of yourself', 'having tubes in your nose or mouth'-'fear of death' [2 items sharing rank 4], 'not being able to sleep'-'not knowing where you are' [2 items sharing rank 5], 'not having treatments explained to you'-'hearing your heart monitor go off' [2 items sharing rank 6], 'being stuck with needles'-'having your blood pressure taken too often' [2 items sharing rank 7], 'being thirsty'-'only seeing family and friends for a few minutes each day'-'having nurses use words you cannot understand' [3 items sharing rank 8], 'hearing people talk about you'-'unfamiliar and unusual noises'-'having no privacy'-'having strange machines around you'-'hearing other patients cry out'-'financial worries' [6 items sharing rank 9], 'missing your husband or wife'-'being in a room which is too hot or cold'-'having lights on constantly'-'having men and women in the same room'-'not knowing when to expect things to be done'-'fear of AIDS'-'being pressurized to consent to treatments' [7 items sharing rank 10].
Dessotte et al 2016 [48]	**Strongest stressors, mean item scores, P:** 'being thirsty', 'having tubes in your nose or mouth'-'not being able to sleep' [2 items sharing rank 2], 'having to look at the pattern of tiles on the ceiling'-'being unable to fulfil family roles' [2 items sharing rank 3], 'being restricted by tubes/lines'-'missing your husband, wife or companion' [2 items sharing rank 4], 'not being able to move your hands or arms because of intravenous (IV) lines'-'having lights on constantly'-'not being in control of yourself' [3 items sharing rank 5], 'being in pain'-'not being able to communicate'-'not knowing the length of stay in ICU [3 items sharing rank 6], 'only seeing family and friends for a few minutes each day', 'fear of death', 'uncomfortable bed or pillow'-'not knowing when to expect things to be done' [2 items sharing rank 9], 'being stuck with needles'-'not knowing what day it is'-'having no privacy'-'financial worries' [4 items sharing rank 10].
Dias et al 2015 [34]	**Strongest stressors, mean item scores:**

Author, Year Country	Main Results: - Comparison of general stressor rating intensities of Ps, HCPs and Rs - Ranking list of strongest stressors, starting with rank 1; up to ten ranks [a)]
	**P, coronary ICU:** 'being in pain', 'being unable to fulfil family roles', 'being bored', 'not being able to sleep', 'financial worries', 'not being in control of yourself', 'not being able to communicate', 'hearing people talk about you'-'fear of AIDS' [2 items sharing rank 8], 'having no privacy', 'only seeing family and friends for a few minutes each day', 'missing your husband or wife' [2 items sharing rank 10]. **P, postoperative ICU:** 'being in pain'-'being unable to fulfil family roles' [2 items sharing rank 1], 'not being able to communicate', 'not being able to sleep'-'fear of AIDS' [2 items sharing rank 3], 'having no privacy', 'being bored', 'being in a room which is too hot or cold', 'having lights on constantly', 'not being able to move your hands or arms because of intravenous lines', 'being thirsty'-'financial worries' [2 items sharing rank 9], 'being stuck with needles'-'not being in control of yourself' [2 items sharing rank 10].
Granja et al 2005 [5]	**Strongest stressful experience, percent stressful recollection, P:** 'tracheal tube aspiration', 'nose tube', 'family worries', 'pain'-'immobilization in bed'-'fear of dying or uncertainty about the future' [3 items sharing rank 4], 'daily needle punctures', 'communication difficulties', 'machine (ventilator) dependence'-'general discomfort' [2 items sharing rank 7], 'urinary catheter', 'noisy and non-sleeping nights', 'lack of privacy in hygiene'.
Gültekin et al 2018 [49]	**Strongest stressors, mean item scores, P:** 'being thirsty', 'having tubes in your nose or mouth', 'not being in control of yourself', 'not being able to sleep', 'being in pain', 'not being able to move your hands or arms because of intravenous (IV) lines', 'only seeing family and friends for a few minutes each day', 'being in a room which is too hot or cold', 'having lights on constantly', 'having to look at the pattern of tiles on the ceiling'.
Heidemann et al 2011 [50]	**Strongest stressors, P, sum of day 1, 2, 3:** 'missing your husband or wife', 'not being in control of yourself', 'being in pain'-'not being able to move your hands or arms because of intravenous (IV) lines'-'being stuck with needles'-'only seeing family and friends for a few minutes each day' [4 items sharing rank 3], 'not being able to sleep'-'having lights on constantly' [2 items sharing rank 4], 'not knowing when to expect things to be done'-'not knowing what time it is' [2 items sharing rank 5], 'having to look at the pattern of tiles on the ceiling'-'having to wear oxygen' [2 items sharing rank 6]; *the remaining 29 items share rank 10 rated as 'not stressful'.*

Author, Year Country	Main Results: - Comparison of general stressor rating intensities of Ps, HCPs and Rs - Ranking list of strongest stressors, starting with rank 1; up to ten ranks [a)]
Hweidi 2007 [51]	**Strongest stressors, mean item scores, P:** 'having tubes in your nose or mouth', 'being in pain', 'not being able to sleep', 'hearing buzzers and alarms from the machinery', 'being thirsty', 'not being in control of yourself', 'unfamiliar and unusual noises', 'being tied down by tubes', 'watching treatments being given to other patients', 'being awakened by nurses'.
Hweidi & Nizamli 2015 [52]	**Strongest stressors, mean item scores, P:** 'being in pain', 'not being able to sleep', 'not being in control of yourself', 'having tubes in your nose or mouth', 'being thirsty'.
Kalfon et al 2010 [6]	**Strongest stressors, mean item scores, P:** 'sleep deprivation', 'perfusion lines, connecting wires, and cables', 'pain'-'thirst' [2 items sharing rank 3], 'noise'-'anxiety' [2 items sharing rank 4], 'limited visiting hours'-'absence of phone' [2 items sharing rank 5], 'bed-related discomfort', 'lack of information', 'excessive light', 'feeling of cold'-'isolation' [2 items sharing rank 9], 'feeling of heat'.
Locihova et al 2018 [35]	**General stress ratings:** P < HCP< R; p<0.001 for P<HCP and P<R; no information on p for HCPR<R<br>**Strongest stressors, mean item scores:** [b)]<br>**P:** 'being in pain', 'being in a room which is too hot or cold', 'fear of death', 'financial worries'-'having tubes in your nose or mouth' [2 items sharing rank 4], 'missing your husband or wife', 'hearing other patients cry out'-'being unable to fulfil family roles' [2 items sharing rank 6], 'not being able to sleep', 'uncomfortable bed or pillow', 'having your blood pressure taken often', 'not being in control of yourself'.<br>**R:** 'being in pain'-'fear of death' [2 items sharing rank 1], 'financial worries', 'being unable to fulfil family roles'-'not being able to sleep'-hearing other patients cry out' [3 items sharing rank 3], 'being in a room which is too hot or cold', 'having the nurses be in too much of a hurry'-'uncomfortable bed or pillow'-'being thirsty' [3 items sharing rank 5], 'missing your husband or wife', 'not knowing what time it is', 'being restricted by tubes/lines'-'not having the nurses introduce themselves'-'not knowing what day it is [3 items sharing rank 8], 'having tubes in your nose or mouth'-'having men and women in the same room', [2 items sharing rank 9], 'having nurses use words you cannot understand'.<br>**HCP:** 'fear of death', 'having tubes in your nose or mouth', 'being in pain', 'hearing other patients cry out'- 'not being in control of yourself'-'not being able to sleep' [2 items sharing rank 5], 'being thirsty', 'having your blood pressure taken often', 'being in a room which is too hot or cold'-'not

Author, Year Country	Main Results: - Comparison of general stressor rating intensities of Ps, HCPs and Rs - Ranking list of strongest stressors, starting with rank 1; up to ten ranks [a)]
	being able to communicate' [2 items sharing rank 8], 'uncomfortable bed or pillow', 'having to look at the pattern of tiles on the ceiling'-'being aware of unusual smells around you' [2 items sharing rank 10].
Mistraletti et al 2018 [36]	**General stress ratings,** P < R < HCP; p<0.05 for P<HCP and P<R_1, R_2; both significant and nonsignificant p-values for $R_{1,2}$ < HCPR, depending on scales of item domains (environment, relation-ship, emotional, physical): **Strongest stressors, mean item scores of product 'intensity by frequency ratings':** [c)] **P:** 'having tubes in nose/mouth which limit movements', 'not being able to speak / express discomfort', 'not being able to move as you wish', 'being thirsty', 'not being able to sleep', 'having to look at ceiling with lights always on', 'missing your loved ones', 'not knowing what day it is', 'being worried/scared', 'having blood pressure measured many times per day', 'feeling pain' [2 items sharing rank 10]. **R_1:** 'having tubes in nose/mouth which limit movements', 'not being able to move as you wish', 'being worried/scared', 'not being able to speak / express discomfort', 'not being in control of yourself', 'feeling pain', 'having to look at ceiling with lights always on', 'not being able to sleep', 'not knowing what day it is', 'being thirsty'. **R_2:** 'having tubes in nose/mouth which limit movements', 'not being able to move as you wish', 'not being able to speak / express discomfort', 'being worried/scared', 'missing your loved ones', 'having unfamiliar devices or iv bags around the body', 'not being in control of yourself', 'having to look at ceiling with lights always on', 'not being able to sleep', 'feeling pain'. **HCP:** 'not being able to speak / express discomfort', 'having tubes in nose/mouth which limit movements', 'not being in control of yourself', 'not having privacy', 'being worried/scared', 'hearing strange / unusual noise', 'being thirsty', 'not being able to move as you wish', 'having to look at ceiling with lights always on', 'feeling pain'.
Nelson et al 2001 [53]	**Strongest stressful experiences, most frequently reported:** **P, % severe and moderate stress:** 'discomfort', 'inability to communicate', 'unsatisfied thirst', 'difficulty sleeping', 'anxiety', 'pain', 'unsatisfied hunger', 'sleep disruption', 'depression', 'shortness of breath'.

Author, Year<br>Country	Main Results:<br>- Comparison of general stressor rating intensities of Ps, HCPs and Rs<br>- Ranking list of strongest stressors, starting with rank 1; up to ten ranks [a)]
Novaes et al 1999<br>[8]	**General stress ratings:** P < R < HCP; p=0.018 for P<HCP; n.s. for P<R and R<HCP<br>**Strongest stressors, mean item scores:**<br>**P:** 'being in pain', 'not being able to sleep', 'having tubes in your nose or mouth', 'not being in control of yourself', 'being tied down by tubes', 'not having treatments explained to you', 'not being able to move your hands or arms because of intravenous (IV) lines', 'not knowing when to expect things to be done', 'being stuck with needles', 'being thirsty'.<br>**R:** 'being in pain', 'having tubes in your nose or mouth', 'being tied down by tubes', 'not being able to sleep', 'not being able to move your hands or arms because of intravenous (IV) lines', 'not being in control of yourself', 'hearing other patients cry out', 'not knowing where you are', 'only seeing family and friends for a few minutes each day', 'missing your husband or wife'.<br>**HCP:** 'being in pain', 'having tubes in your nose or mouth', 'being tied down by tubes', 'not being able to sleep', 'hearing other patients cry out', 'being in a room which is too hot or cold' 'being stuck with needles', 'hearing the buzzers and alarms from the machinery', 'not having treatments explained to you', 'hearing your heart monitor alarm go off'.
Pagnucci et al 2019<br>[54]	**Strongest stressors, most frequently reported:**<br>**P, % 'a lot stressful':** 'to be thirsty', 'health care staff that talk, joke and discuss issues in loud voices', 'being worried / afraid', 'seeing healthcare profes-sionals too busy, stressed or hurried', 'hearing other patients suffering, crying or complain', 'being awakened by healthcare professionals', 'having needles inserted', 'not being able to freely take the position desired - having tubes in the nose or mouth that restrict movement' [2 items sharing rank 8], 'not being able to sleep', 'to have pain - having the sensation of not eating' [2 items sharing rank 10].
Paldon et al 2014<br>[18	**General stress ratings:** P < HCP, p<0.001<br>**Strongest stressors, mean item scores:**<br>**P:** 'not being able to sleep', 'financial worries', 'not being able to fulfil family responsibilities', 'being in pain', 'being pricked by needles frequently'<br>Stressor intensity: Physical > psychological > environmental, no data on p.

Author, Year Country	Main Results: - Comparison of general stressor rating intensities of Ps, HCPs and Rs - Ranking list of strongest stressors, starting with rank 1; up to ten ranks [a]
	**HCP:** 'fear of death', 'being in pain', 'not being able to fulfil family responsibilities', 'financial worries', 'being pricked by needles frequently'<br>Stressor intensity: Physical > psychological > environmental, no data on p.
Pang & Suen 2008, 2009 [37, 42]	**General stress ratings:** P < HCP, p<0.001; P < R, p<0.001; no data on the comparison HCP versus R.<br>**Strongest stressors, mean item scores:**<br>**P:** 'fear of death', 'being pressurized to consent to treatment', being in pain', 'not knowing the length of stay in ICU', 'not being able to communicate', 'fear of hospital-transmitted diseases',[d] 'not having treatments explained to you', 'financial worries', 'having tubes in your nose or mouth', 'unfamiliar and unusual noises'.<br>**R:** 'fear of death', 'being in pain', 'not being in control of yourself', 'not knowing the length of stay in ICU', 'having tubes in your nose or mouth', 'being stuck with needles', 'not being able to communicate', 'hearing your heart monitor alarm go off', 'not having treatments explained to you', 'being restricted by tubes/lines'.<br>**HCP:** 'fear of death', 'not being in control of yourself', 'not being able to communicate', 'being pressurized to consent to treatment', 'having tubes in your nose or mouth', 'being in pain', 'hearing the buzzers and alarms from the machinery', 'being stuck with needles', 'hearing your heart monitor alarm go off', 'having strange machines around you'.
Patacky et al 1985 [55]	**Strongest stressors, mean item scores:**<br>**P:** 'being admitted to the ICU', 'being unable to move freely in bed because of equipment'-'not knowing or understanding your illness and its seriousness' [2 items sharing rank 2], 'pain and discomfort anticipated as the result of treatments and unexplained procedures', 'being examined by several different doctors and nurses who ask the same questions', 'losing contact with the outside world, dates, and time of day', 'having an intravenous line in your arm', 'lack of personal privacy', 'frequent drawing of blood'-'frequent interruptions of your sleep'-'emergency measures being performed' [3 items sharing rank 8], 'the noise around you', 'limits on visiting by your family and friends'.
Pennock et al 1994 [7]	**Strongest stressors, mean item scores, P:** 'endotracheal tube', 'inability to talk', 'confusion', 'sleeplessness', 'sore throat', 'doctors/nurses talking', 'chest tube removal', 'pain', 'fear of dying', 'inability to eat'.

Author, Year Country	Main Results:<br>- Comparison of general stressor rating intensities of Ps, HCPs and Rs<br>- Ranking list of strongest stressors, starting with rank 1; up to ten ranks [a)]
Pieris et al 2018 [19]	**Strongest stressors, most frequently reported, P:** 'being bedridden', 'pain', 'family worries', 'daily needle punctures', 'general discomfort', 'fear of dying and uncertainty of the future'', 'urinary catheter', 'economic worries', 'dependence on doctors and nurses', 'nose tube'.
Rattray et al 2010 [12]	**Strongest stressful experiences, most frequently reported, P:** 'most of my memories are blurred', 'I never knew whether it was day or night', 'I felt helpless', 'I thought I would die', 'I wish I had known more about what was'-'I wish I remembered more about it' [2 items sharing rank 5], 'I seemed to sleep too much', 'I felt scared', 'I had bad dreams', 'I was constantly disturbed', 'it was always too noisy'-'I saw strange things' [2 items sharing rank 10].
Rosa et al 2008 [56]	**Strongest stressors, mean item scores, P:** 'being in pain', 'not being able to eat', 'being unable to fulfil family roles', 'not knowing the length of stay in ICU', 'having lights on constantly', 'being stuck with needles', 'being thirsty', 'not being able to move your hands or arms because of intravenous (IV) lines', 'having no privacy', 'financial worries'. *Note: Item 'being unable to eat' seems not to belong to original ESQ*
Rotondi et al 2002 [1]	**Strongest stressors out of ICU stressors, most frequently reported, P:** 'spell of terror or panic', 'nightmares', 'pain', 'being restrained', 'feeling something bad will happen', 'not being able to sleep', 'not being in control', 'trouble speaking', 'difficulty swallowing', 'being thirsty'.<br>**Strongest ETT experiences, most frequently reported, P:** 'not getting enough air from ETT', 'feeling anxiety about ETT', 'feeling choked by ETT', 'pain or discomfort associated with ETT', 'not being able to speak'.
Rowe & Weinert 1987 [57]	**Strongest stressors, mean item scores, P:** 'thinking you might be having a heart attack', 'having a sudden hospitalization you weren't planning to have', 'not being told what your diagnosis is', 'thinking you might lose your sight', 'not knowing the results or reasons for your treatments', 'knowing that you have a serious illness', 'having nurses or doctors talk too fast or use words you can't understand', 'not knowing for sure what illness you have', 'missing your spouse', ''having to be assisted with the bedpan'.
Sharmila et al 2016 [38]	**General stress ratings:** authors report P> HCPs; adequate information on statistical significance tests is lacking.<br>**Strongest stressors, no data on ranking procedure:**

**Author, Year Country**	**Main Results: - Comparison of general stressor rating intensities of Ps, HCPs and Rs - Ranking list of strongest stressors, starting with rank 1; up to ten ranks [a)]**
	**P:** 'being restricted by tubes/lines', 'fear of death', 'being in pain', 'financial worries', 'being unable to fulfil family roles', 'being stuck with needles', 'not knowing the length of stay in ICU', 'having to wear oxygen' - 'missing your husband or wife' [2 items sharing rank 8], 'being in a room which is too hot or cold', 'having tubes in your nose or mouth'. **HCP:** 'being restricted by tubes/lines', 'fear of death', 'being in pain', 'being unable to fulfil family roles', 'being stuck with needles' / 'financial worries' [2 items sharing rank 4], 'missing your husband or wife', 'not knowing the length of stay in ICU', 'having strange machines around you' - 'having to wear oxygen' [2 items sharing rank 8], 'not being able to move your hands or arms because of intravenous (IV) lines', 'only seeing family and friends for a few minutes each day'.
So & Chan 2004 [39]	**General stress ratings:** data of specific stressors indicate P<HCPs; adequate information on statistical significance tests is lacking. **Strongest stressors, mean item scores:** **P:** 'being tied down by tubes', 'not being in control of yourself', 'not being able to sleep', 'hearing the buzzers and alarms from the machinery', 'being thirsty', 'being in pain' - 'not knowing when to expect things will be done to you' [2 items sharing rank 6], 'having your blood pressure taken often', 'missing your husband or wife' - 'having the nurses be in too much of a hurry' - 'not being able to move your hands or arms because of intravenous (IV) lines' [3 items sharing rank 8], 'having tubes in your nose or mouth' - 'having to look at the pattern of tiles on the ceiling' [2 items sharing rank 9], 'seeing intravenous (IV) bags over your head' - 'frequent physical exams by doctors and nurses' [2 items sharing rank 10]. **HCP:** 'being tied down by tubes', 'being stuck with needles', 'having tubes in your nose or mouth', 'hearing the buzzers and alarms from the machinery', 'having strange machines around you', 'being in pain', 'not knowing when to expect things will be done to you' - 'hearing your heart monitor alarm go off' [2 items sharing rank 7], 'not being in control of yourself', 'not having treatments explained to you', 'not being able to sleep'.
Soehren 1995 [24]	**Strongest stressors, mean item scores, P:** 'having tubes in your nose or mouth', 'being tied down by tubes', 'being in pain', 'not being in control of yourself', 'missing your husband or wife', 'not being able to sleep', 'being thirsty', 'being stuck with needles', 'not being able to move your hands or arms because of intravenous (IV) lines', 'being in a room which is too hot or cold' [2 items sharing rank 9], 'being awakened by nurses', 'having lights on constantly' [2 items sharing rank 10].

Author, Year Country	Main Results: - Comparison of general stressor rating intensities of Ps, HCPs and Rs - Ranking list of strongest stressors, starting with rank 1; up to ten ranks [a)]
Soh et al 2008 [58]	**Strongest stressors, mean item scores P:** 'being in pain', 'being stuck with needles', 'being bored', 'missing your husband or wife' - 'being in a room which is too hot or cold', [2 items sharing rank 4], 'not being able to sleep', 'not being able to move your hands or arms because of intravenous (iv) lines', 'having tubes in your nose or mouth', 'having to look at the pattern of tiles on the ceiling', 'being thirsty', 'financial worries'.
Soh et al 2014 [59]	**Strongest stressors, mean item scores, P:** 'tracheal tube aspiration' (endotracheal suctioning), 'pain', 'bedridden', 'general discomfort', 'ventilator dependence', 'daily needle punctures'.
Takashima et al 2018 [60]	**Strongest stressors out of ICU and ETT stressors, mean item scores, P:** 'being thirsty', 'not being able to speak' - 'pain or discomfort associated with ETT [2 items sharing rank 2], 'not being able to move freely because of the tube and infusion', 'having difficulty talking', 'ETT was uncomfortable', 'aspiration was hard', 'pain', 'I felt tense', 'not being able to control myself', 'I felt scared'.
Turner et al 1990 [61]	**Strongest stressors, mean item scores, P:** 'arterial blood gas sampling', 'tracheal suctioning', 'pain', 'noise', 'family worries', 'frustration', 'hunger', 'alarms', 'fear of procedures', 'financial worries'.
Wilson 1987 [62]	**Strongest stressors, P, mean item score:** 'having pain' - 'not being able to move freely' [2 items sharing rank 1], 'frequent interruptions of sleep', 'being thirsty' - 'having too many tubes' [2 items sharing rank 3], 'having major surgery', 'being on ventilator or having oxygen', 'frequent drawing of blood', 'having needle (IV) in your arm', 'losing track of time', 'uncomfortable temperature', 'having doctors and nurses talk about you rather than to you'.
Yava et al 2010 [40]	**General stress ratings:** P < HCP, p<0.001. **Strongest stressors, mean item scores:** **P:** 'fear of death', 'being thirsty', 'being in pain', 'not being able to sleep', 'having tubes in your nose or mouth', 'hearing other patients cry out', 'being restricted by tubes/lines', 'not being able to move your hands or arms because of intravenous (IV) lines', 'uncomfortable bed or pillow', 'having lights on constantly'.

Author, Year Country	Main Results: - Comparison of general stressor rating intensities of Ps, HCPs and Rs - Ranking list of strongest stressors, starting with rank 1; up to ten ranks [a)]
	**HCP:** 'fear of death', 'being in pain', 'having tubes in your nose or mouth', 'not being able to sleep' - 'having no privacy', 'not knowing the length of stay in ICU' [2 items sharing rank 5], 'hearing your heart monitor alarm go off', 'hearing other patients cry out', 'not being able to communicate, 'not being in control of yourself', 'being restricted by tubes/lines'.
Zengin et al 2020 [63]	**Strongest stressors (sum of % of 'a lot of stress', 'some stress'), P:** 'thirst', 'pain', 'separation from family', 'inability to sleep', 'endotracheal tube aspiration', 'loneliness', 'presence of tubes in the nose' - 'mechanical ventilation' [2 items sharing rank 7], 'inability to speak', 'oxygen mask / tube', 'noise (e.g. noise from machines)'.

[a)] ranking lists contain a minimum of 5 stressors, and a maximum of 10 ranks. In case of shared ranks, more than 10 stressors are included to reach the maximum number of 10 ranks (details in methods section 'extraction and management of data'); [b)] data obtained from first author upon request; [c)] formulation of several items is modified to an extent that makes comparison with original ICUESS items impossible; [d)] the original item 'fear of AIDS' was modified to 'fear of other hospital-transmitted diseases'. Abbreviations: **ETT:** endotracheal tube; **HCP**: Health care providers; **ICU:** Intensive care unit; **n. s.:** not significant; **P**: Patient; **R**: Relatives.

Supplementary Table S2: Synthesis of most severe perceived stressors in the ICU, patient ratings, sorted in alphabetic order; source: 42 original studies from 1981 to 2020 +

**Domain: Physical, treatment and disease-related stressors, 46 items**
'bed-related discomfort', 'being admitted to the ICU', 'being awakened by nurses', 'being bedridden', 'being in pain', 'being restricted by tubes/lines', 'being stuck with needles', 'being thirsty', 'being unable to move freely in bed because of equipment', 'chest tube removal', 'coughing while receiving mechanical ventilation', 'daily needle punctures', 'difficulties in sleeping', 'difficulty swallowing', 'emergency measures being performed', 'endotracheal tube aspiration / suctioning (ETT)', , 'feeling of cold', 'feeling of heat', 'frequent drawing of blood', 'frequent interruptions of sleep', 'frequent physical exams by doctors and nurses', 'having a sudden hospitalization you weren't planning to have', 'having major surgery', 'having the sensation of not eating', 'having to be assisted with the bedpan', 'having tubes in your nose and/or mouth', 'having to wear an oxygen mask', 'having your blood pressure taken often', 'hearing your heart monitor alarm go off', 'hunger', 'I seemed to sleep to much', 'machine (ventilator) dependence', 'not being able to eat', 'not being able to go to the bathroom', 'not being able to move as you wish', 'not being able to move freely', 'not being able to sleep', 'pain and discomfort anticipated as the result of treatments and unexplained procedures', 'pain or discomfort associated with ETT', 'secretion', 'shortness of breath', 'sleeplessness', 'sleep deprivation', 'sore throat', 'seeing intravenous (IV) bags over your head', 'urinary catheter'.
**Domain: Mental health, 55 Items**
'agony/panic', 'anxiety/fear', 'being bored', 'being unable to fulfil family roles', 'being worried/scared', 'concerns about their health and its impact on their daily life', 'concern for family', 'confusion', 'depression', 'family worries', 'fear of AIDS', 'fear of death', 'fear of hospital-transmitted diseases', 'fear of medical procedures',

'feeling anxiety about ETT', 'feeling something bad will happen', 'financial worries', 'frustration', 'general discomfort, 'having no privacy', 'I had bad dreams', 'I saw strange things', 'I felt tense', 'I felt helpless', 'I felt scared', 'I thought I would die', 'I was constantly disturbed', 'I wish I had known more about what was happening to me', 'I wish I remembered more about it', 'insecurity', 'isolation', 'knowing that you have a serious illness', 'limits on visiting by your family and friends', 'loneliness', 'losing contact with the outside world, dates, and time of day', 'losing track of time', 'missing your loved ones / family and friends', 'missing your spouse', 'most of my memories of intensive care were blurred', 'my care could have been better', 'my memories in intensive care are frightening', 'nightmares', 'not being able to fulfil family responsibilities', 'not being in control of yourself', 'not having control over their activities', 'not knowing the length of stay in ICU', 'not knowing what day it is', 'not knowing what time it is', 'not knowing where you are', 'only seeing family and friends for a few minutes each day', 'separation from family', 'spell of terror or panic,' 'thinking you might be having a heart attack', 'thinking you might lose your sight', uncertainty about the future'.
**Domain: Communication, 21 Items**
'being examined by several different doctors and nurses who ask the same questions', 'being pressurized to consent to treatment', 'communication difficulties', 'dependence on doctors and nurses', 'doctors/nurses talking', 'feeling the nurses are watching the machines closer than they are watching you', 'having doctors and nurses talk about you rather than to you', 'having nurses or doctors talk too fast or use words you can't understand', 'having the nurse / health care professionals be in too much of a hurry', 'health care staff that talk, joke and discuss issues in loud voices', 'hearing people talk about you', 'lack of information', 'not being able to communicate', 'not being able to talk', 'not being told what your diagnosis is', 'not having treatments explained to you', 'not knowing for sure what illness you have', 'not knowing or understanding your illness and its seriousness', 'not knowing the results or reasons for your treatments', 'not knowing when to expect things to be done', 'not knowing when to expect things will be done to you'.

**Domain: Environment, 15 Items**
'absence of phone', 'alarms', 'being in a room which is too hot or cold', 'excessive light', 'having lights on constantly', 'having to look at the pattern of tiles on the ceiling', 'hearing buzzers and alarms from the machinery', 'hearing other patients cry out', 'it was upsetting to see what happened to other patients', 'limited visiting hours', 'noise from machines', 'the noise around you', 'uncomfortable bed or pillow', 'unfamiliar and unusual noises', 'watching treatments being given to other patients'.

+ Details see supplementary Table 1
